# Protocol for detecting unrecognized sleep apnea in patients with atrial fibrillation by a home-monitoring device: the DAN-APNO study

**DOI:** 10.1186/s12872-021-02453-0

**Published:** 2022-01-08

**Authors:** Mads Hashiba Jensen, Frederik Dalgaard, Rasmus Rude Laub, Vibeke Gottlieb, Morten Lock Hansen, Olav Vendelboe, Jim Hansen, Morten Lamberts

**Affiliations:** 1 Department of Cardiology, Copenhagen University Hospital Herlev and Gentofte, Kildegaardsvej 28, 2900 Hellerup, Denmark; 2 Department of Pulmonology, Herlev and Gentofte Hospital, Copenhagen, Denmark; 3 Department of Cardiology, Bispebjerg Hospital, Copenhagen, Denmark

## Abstract

**Background:**

Determining the presence of modifiable risk factors for atrial fibrillation (AF), such as sleep apnea is of clinical importance due to the potential impact targeting these risk factors can have on the progression and burden of AF. Using new digital-based technology is a promising solution to the underreporting of sleep apnea highlighted by academical societies in recent years. The aim of this study is to report the prevalence and severity of sleep apnea in patients with AF and, secondarily, assess the accuracy and feasibility of a new home-screening device for sleep apnea (NightOwl™ by Ectosense).

**Methods:**

DAN-APNO is a cross-sectional study at the Department of Cardiology, Herlev-Gentofte Hospital recruiting patients with AF referred to anticoagulation initiation aged 18 to 90 years without known sleep apnea. At least 150 patients will be recruited and undergo medical history, clinical evaluation, several sleep-apnea questionnaires, and a sleep-recording evaluation for four nights with sleep apnea home-monitoring device NightOwl™. Additionally, the first 20 participants and participants with moderate-severe sleep apnea by screening are referred to cardio-respiratory monitoring (CRM). This clinical evaluation allows the comparison of standard evaluation method and the NightOwl™. Clinical measures include Apnea–Hypopnea Index (AHI), Oxygen Desaturation Index (ODI), pulse rate, as well as questionaries about sleep apnea assessment and the clinical feasibility of the NightOwl™ device. Main outcomes comprise analysis of the prevalence and severity of sleep apnea, and clinical and demographic predictors of moderate and severe sleep apnea. In addition, correlation analyses for accuracy measures between CRM and NightOwl™ will be conducted along with patient ease-of-use and satisfaction questionnaires.

**Discussion:**

This study is limited by selection bias; only patients with atrial fibrillation from anticoagulation clinic is asked to participate, which could limit the generalizability of our results. However, this study aims to test whether a miniaturized simple home-monitoring device for detecting sleep apnea in patients with AF potentially can evaluate sleep apnea more conveniently and easier.

*Trial Registration* The study is registered the 18-02-2021 at clinicaltrials.gov with registration number: NCT04760002.

## Strengths and limitations of this study

- This study aims to test whether a miniaturized simple home-monitoring device for detecting sleep apnea in patients with atrial fibrillation potentially can evaluate sleep apnea more conveniently and easier.
- It will simultaneously gather extensive clinical characteristics, hoping to be able to identify important predictors for sleep apnea in patients with atrial fibrillation and evaluate the usefulness of implemented questionnaires when screening and evaluating symptom burden for sleep apnea.
- This study is limited by selection bias; only patients with atrial fibrillation from anticoagulation clinic is asked to participate, which could limit the generalizability of our results.
- This study does not assess the prevalence of sleep apnea by gold standard of polysomnography (PSG) but by home-monitoring device and CRM, therefore there may be higher risk of false negative and false positives.

## Background

Sleep apnea refers to intermittent, cyclical cessations or reductions of airflow and is characterized by snoring, oxygen desaturations and brief arousal from sleep resulting in frequent interruption of respiration and sleep. The most prevalent form is obstructive sleep apnea (OSA) with partial or complete collapse of the upper airway, and this form will throughout this protocol be referred to as sleep apnea. The last two decades, OSA has been recognized as an increasingly common condition occurring in up to 17% of males and 10% of females aged between 50 and 70 years [1, 2]. OSA is also a known risk factor for developing and exacerbating multiple cardiovascular diseases including atrial fibrillation (AF) [3–8]. The diagnosis of sleep apnea is typically based on the number of apnea and hypopnea per hour of sleep, titled the Apnea–Hypopnea Index (AHI).

In patients with AF, OSA has been shown to be associated with a higher risk of recurrent AF episodes after procedures restoring the heart rhythm, such as catheter ablation and electrical cardioversion [9, 10]. There is also evidence that OSA are related to the progression of AF [11], have been shown to reduce the effectiveness of rhythm drug therapy, [12] and associated with increased risk of cardiovascular events such as stroke [13]. In total, presence of concomitant OSA and AF is assumed to constitute a higher cardiovascular disease burden and worse symptoms and related to poorer prognosis. OSA is a modifiable risk factor, that can be treated with weight loss, oral appliances, continuous positive airway pressure (CPAP) and surgery. However, randomized clinical trial are yet to establish benefits of sleep apnea treatment in these patients.

To determine presence of sleep apnea patients with AF is therefore clinically important but is often overlooked and the diagnostic work-up is cumbersome, time-consuming and not widely available. The gold standard of sleep apnea diagnosis and AHI classification is a polysomnography (PSG) but sleep apnea is routinely diagnosed with cardiorespiratory monitoring (CRM) [14]. Academical societies have in recent decades advocated that OSA is underreported and unrecognized, and increasing evidence is supporting this statement. In a recent investigation of 1364 patients without OSA undergoing non-cardiac surgery, researchers found a prevalence of 67.6% with 11.2% suffering from severe OSA [15]. Another study of patients with AF (n = 41) the prevalence of OSA was 56% [16], and in similar study of patients with AF (n = 100), 85% had OSA (AHI ≥ 5) and severe OSA was detected in 28% [17].

A common and important clinical consequence of sleep apnea is excessive daytime sleepiness, and this clinical consequence is advocated by the use of questionnaires to assess daytime sleepiness. However, studies have shown most patients with AF report low daytime sleepiness although subsequently polysomnography analysis graded by AHI found sleep apnea present [18]. As such, screening and management of sleep apnea in patients with AF requires new prediction and assessment tools. Possibly, emerging new technologies consisting of miniaturized easily available monitoring devices and accurate algorithm development may help in the evaluation of sleep apnea status in the large number of patients with AF [19]. One of such devices is the NightOwl™, a self-applied home-monitoring device made to detect sleep apnea. It has been validated against PSG in a single-night in-laboratory setting showing promising results but a multi-night assessment of NightOwl™ in a home environment is still to be examined [20]. Potentially, the device could be an easy and inexpensive method to find undiagnosed sleep apnea in patients with AF.

## Objectives and hypothesis

To use a home-monitoring device NightOwl™ to screen patients with AF for sleep apnea and thus;

1. Report the prevalence and severity of sleep apnea in patients with AF.
2. Assess the accuracy, user-experience, and feasibility of home-screening for sleep apnea in patients with AF.

### **Hypothesis**

In patients with AF, the prevalence of unrecognized OSA is high (around 50%) and home-monitoring test using NightOwl™ is a viable, easy, and accurate test of measuring sleep apnea.

## Method and design

### Setting

Patients diagnosed with any type of AF referred to anticoagulation initiation at a nurse-run ambulatory will be asked to participate. The ambulatory consists of four daily nurse-led tracks at Department of Cardiology, Herlev-Gentofte University Hospital. In a formal collaboration, Department of Pulmonology, Herlev-Gentofte University Hospital provides work-up with CRM investigation and clinical evaluation of initiating treatment of sleep apnea in patients referred from the study.

### Participants

Participants with AF, without known sleep apnea, and indication for anticoagulation will be recruited from the Thrombosis unit. Inclusion and exclusion criteria are shown in Box 1. The following information will be obtained from the participants:

- Clinical data captured at first visit: age, sex, body mass index, neck circumference and blood pressure.
- Data captured from electronic health system: latest available blood samples, prior health conditions (comorbidities), prior procedures performed such as electrocardiography and echocardiogram, and prior procedures related to AF such as electrical cardioversion and ablation.
- Questionnaires for sleep apnea screening and symptomatic burden of sleep apnea: STOP-BANG, Berlin Questionnaire and Epworth Sleepiness Scale (ESS).

**Box 1:** Inclusion and exclusion criteria

Inclusion criteria	Exclusion criteria
Diagnosis of AF of any type	Known sleep apnea
Age < 90 years	Secondary AF
Age > 18 years	Professional drivers
	Severe heart failure (NYHA class III or IV)
	Severe chronic obstructive pulmonary disease

### Outcomes

The primary outcome of the study is the prevalence with 95% confidence intervals of sleep apnea in patients with AF detected by NightOwl™. This analysis will be total and stratified by severity of sleep apnea.

The secondary outcome will be an exploratory analysis of predictors of sleep apnea and predictors of moderate and severe sleep apnea in the participants using baseline characteristics and clinical data of the participants.

Other outcomes of interest are;

1. The correlation between NightOwl™ and CRM diagnostic testing for sleep apnea and the correlation between sleep apnea screening questionnaire for CRM and NightOwl™.
2. Evaluation of user-experience and compliance with NightOwl™.
  The evaluation of user-experience and compliance will consist of a survey questionnaire build with help from CATCHET Unified Method for Assessment of Clinical Feasibility (CUMACF) [21]. The purpose of this method is to deliver a standardized way of measuring clinical feasibility of the many new technologies being designed and tested. The clinical feasibility questionnaire is divided into three parts: Usage Adoption, Perceived Usefulness and Usability, and Health Efficacy.
3. Inter-variability of NightOwl™ recordings.
  Recordings of four nights will be used to assess any inter-variability between the NightOwl™ recording. Furthermore, the four nights recording can clarify potential night-to-night fluctuations and thereby gives a more precise screening of the presence of sleep apnea.

### Study design

**Fig. 1:**
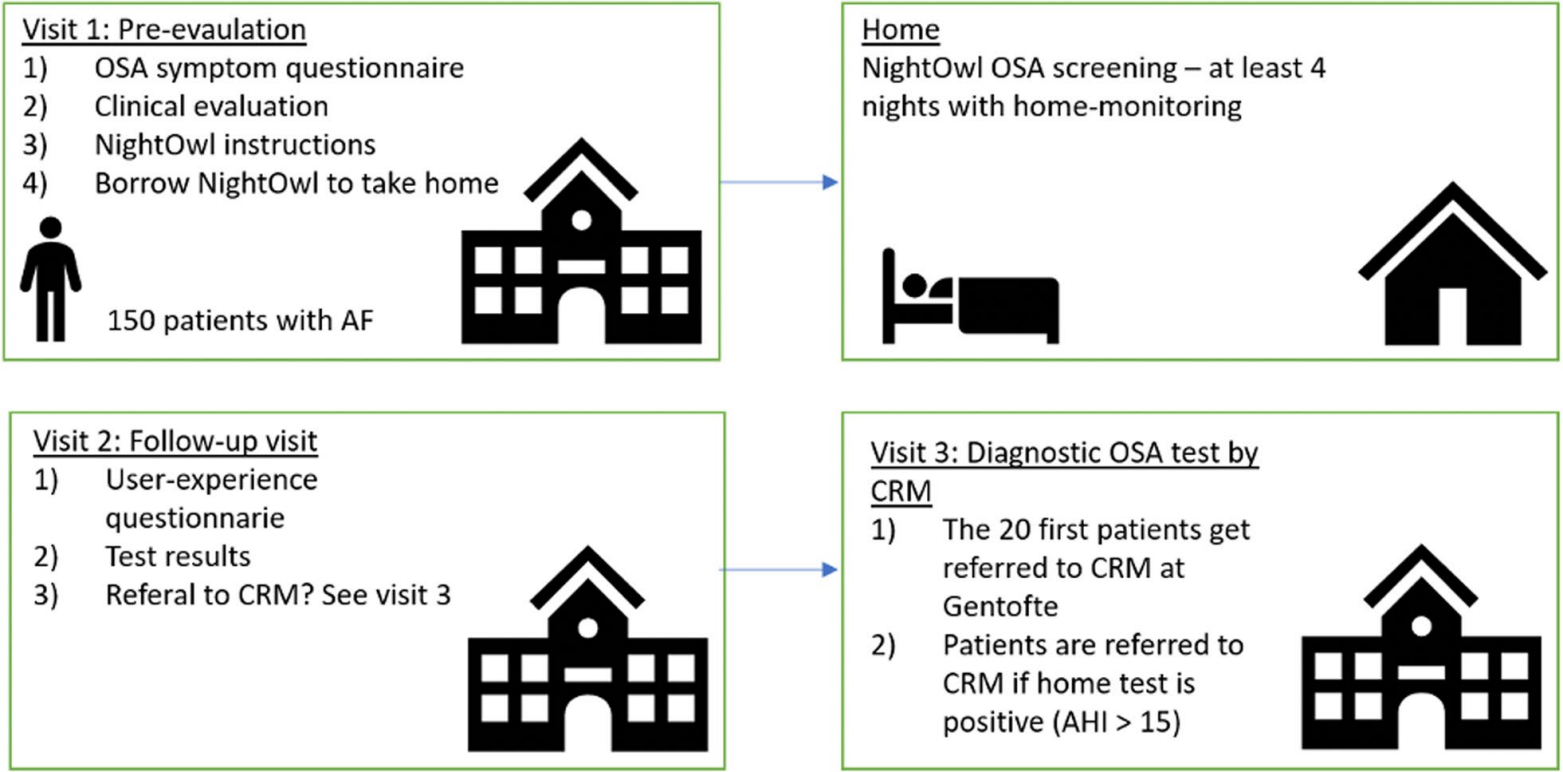
Design of the study

The is a cross sectional study. In total two visits will be planned, with a third visit for the first 20 participants and participants with AHI > 15 included in the study (Fig. 1).

1. Initial visit with time for clinical evaluation, questionnaire of OSA symptoms, the participant borrows a NightOwl™ and receive device instructions.
2. Four night of recording with NightOwl™ in home environment.
3. Follow-up visit for the home-monitoring results and soft node questionnaire.
4. For the 20 first patients and for all patients where the home test is showing (AHI > 15) a fourth visit at the sleep apnea clinic will be arranged.

The time plan for the study inclusion until last patient enrolled are estimated to be approximately 6 months.

### The device

**Fig. 2:**
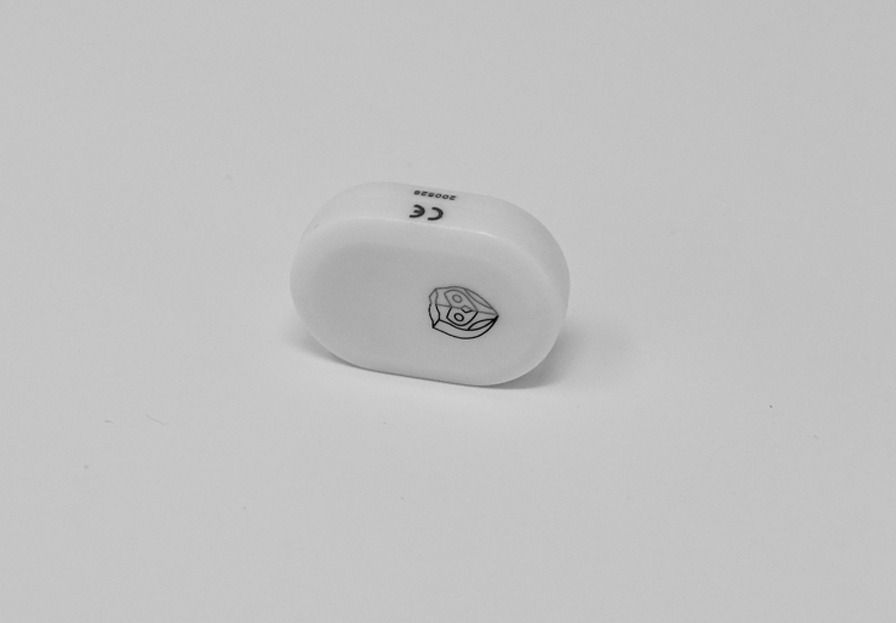
NightOwl™ sensor device

The NightOwl™ consists of a small sensor device which is placed on the fingertip and a smartphone app that is connected to an encrypted cloud-based analytics platform within the European Union, the NightOwl™ software (Fig. 2). It is self-applied by attaching the sensor to the fingertip by means of an adhesive patch. The NightOwl™ sensor acquires accelerometer data and reflectance based photoplethysmography (PPG) from which it derives actigraphy (sleep/wake behavior), saturation of peripheral oxygen (SpO2), peripheral artery tone (PAT) and pulse rate, among other features [20]. A PAT analysis derives changes in caliber of arteries elicited by alterations in the contractile activity of vascular smooth muscle and are referred to as changes in arterial tone. The end state of the apnea–hypopnea events are associated with sympathetic activation [22]. Conversely, this autonomic arousal is also associated with nonrespiratory events e.g. the episodic limb movements during sleep, which could lead to a false-positive respiratory event in the PAT analysis. However, by incorporating concurrent analysis of other PPG-derived features such as SpO_2_ the discrimination amongst type of sympathetic activation associated events are made possible [20].

**Fig. 3:**
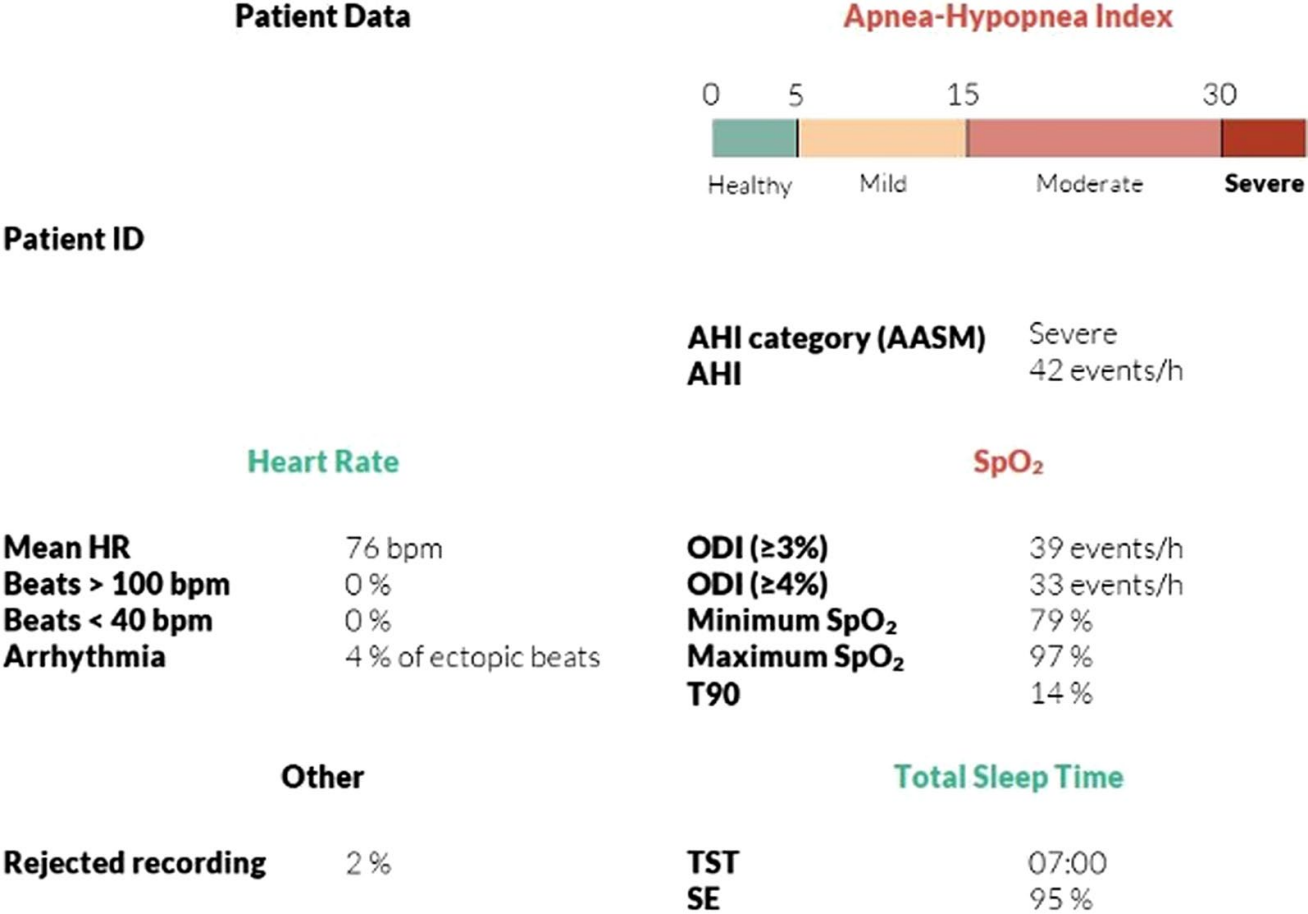
NightOwl™ Data Output (sample). The NightOwl™ derives all parameters sufficient to diagnose sleep apnea

The NightOwl™ software uses an algorithm based on the simultaneous analysis of PPG-derived physiological events, such as PAT, respiratory effort, and SpO2. From these parameters, the software can derive the main clinical parameters used for sleep apnea diagnostics (ODI, AHI among others). As such, the NightOwl™ system can derive all diagnostic parameters recommended by The American Academy of Sleep Medicine Manual for the Scoring of Sleep and Associated Events for home sleep apnea testing [23] (Fig. 3).

The product complies with Medical Device Directive 93/42/ECC and amendments 2007/47/EC requirements. The product is CE marked i.e. the product is authorized for clinical use. Instructions of use and complete product description can be found at http://www.ectosense.com/eifu.

Data captured from NightOwl™ comprises data from four sleep evaluations and consists of the parameters AHI, ODI, heart rate and sleep time. The AHI is graded into four different classifications, them being without sleep apnea (AHI < 5), mild sleep apnea (5 < 15), moderate sleep apnea (15 < 30) and severe sleep apnea (> 30). Thus, NightOwl™ defines sleep apnea as AHI > 5. The data captured from the four nights is to be presented in average quantities thereby counteracting potentially variations in the participants’ sleep data from night to night.

### Cardiorespiratory monitoring

The CRM for the 20 first participants and for the participants where the home test is showing AHI > 15 (moderate-to-severe sleep apnea) is provided by the Department of Pulmonology, Herlev-Gentofte University Hospital and will quantify the frequency, duration and type of each sleep apnea event (obstructive or central) by measuring AHI, ODI, oximetry distribution and heart rate. Potential treatment for sleep apnea will be at the discretion of the attending sleep apnea physician. Simultaneously with undergoing the CRM, the participants will also be assessed by the NightOwl™ the same night. This simultaneity in the recording by the two different monitoring-methods allows for a direct analysis of the correlation of AHI (and other parameters) measured by CRM and NightOwl™.

### Statistical analysis

The prevalence will be calculated by the proportion of participants with sleep apnea out of all participating individuals. The differences between patients with and without a diagnosis of sleep apnea (i.e. AHI > 5) will be assessed by the independent sample t test for continuous variables and the χ2 test for categorical variables. For the participants undergoing CRM, accuracy measurements of the home-monitoring of the NightOwl™ will be conducted by correlation analyses including scatter plots based on AHI measured by CRM versus AHI measured by NightOwl™ and receiver operator characteristics (ROC) with area under the curve (AUC) values.

As an exploratory analysis, a multivariate logistic regression analysis will be performed to test the predictors of a set of factors to detect the presence of any sleep apnea and presence of severe sleep apnea. These include age, body mass index (BMI), sex, neck circumference, European Heart Rhythm Association (EHRA) score (0–4), type of AF (new AF, paroxysmal, persistent AF, permanent AF), prior treatment for AF, blood samples, echocardiographic data and electrocardiogram data. Similar analysis using a multivariate logistic regression will evaluate if commonly used sleep apnea questionnaire can predict presence of sleep apnea in these patients. Correlation analyses will also be conducted of the clinical feasibility questionnaires.

### Power calculations

Assuming a prevalence of severe sleep apnea of 10% in the population compared to 4% in the general population, with alpha 0.05 and a power of 80% a sample size of 113 was calculated. We plan to include 150 patients due to drop-outs, and potentially difficulties of patients applying the device.

### Missing data

Bias due to missing data will be investigated by comparing baseline characteristics of participants with and without missing values. Depending on the extent of missingness, the predictors of missing values will be identified. Multiple imputation using chained equations shall be considered as part of a sensitivity analysis for missing data in the primary outcome model.

## Discussion

This study will estimate the prevalence of unrecognized sleep apnea in patients with AF referred for anticoagulation. Furthermore, we aim to test whether a miniaturized simple home-monitoring device for detecting sleep apnea potentially can evaluate sleep apnea more conveniently and easier. Currently, patients with AF rarely undergo sleep evaluation due to limited resources, lack of standardized evaluation, lack of efficient screening tools, and cumbersome diagnostics. By making it easy and available to evaluate sleep apnea patients with AF, the prevalence of sleep apnea can be determined. Additionally, there is need for better prediction tools and risk scores to identify patients with AF and sleep apnea, by gathering extensive baseline characteristics and clinical data, we hope to be able to identify important predictors for sleep apnea in this population and can evaluate the usefulness of implemented questionnaires in this population when screening and evaluating symptom burden for sleep apnea. In addition, using such a device as the NightOwl™, patients can potentially be more independent of nurse or physician involvement to perform data collection, and clinical assessment and dialogue with patients can be prioritized.

Such cross-sectional studies do not come without limitations. Bias sample selection could apply; only patients with AF from anticoagulation clinic is asked to participate, which could limit the generalizability of our results. We do not assess prevalence of sleep apnea by gold standard of PSG but by CRM, therefore there may be higher risk of false negative and false positives. Using a smart-device such as NightOwl™ also have limitations as the device does not distinguish between central- and obstructive sleep apnea. However, the CRM validation can estimate the prevalence of central- and obstructive sleep apnea in those patients. Additionally, patients can potentially have a high amount of unrecorded data not being able to correctly determine if sleep apnea is present. The four nights of recording should limit this bias. For our secondary outcome of predictors of sleep apnea, we cannot infer causality or temporality since all data is observational and collected at first visit.

## Conclusion

DAN-APNO is a cross sectional study intending to determine the prevalence of unrecognized sleep apnea in patients with AF referred for anticoagulation therapy using a miniaturized easy-to-use tool to diagnose sleep apnea. The results gained from this study will likely improve sleep evaluation and potentially inform future clinical guidelines on prevalence and predictors of sleep apnea in patients with AF.

## Data Availability

Not applicable.

## Abbreviations

AF: Atrial Fibrillation
AUC: Area Under the Curve
AHI: Apnea-Hypopnea Index
BMI: Body Mass Index
CPAP: Continuous positive airway pressure
CRM: Cardio-Respiratory Monitoring
CUMACF: CATCHET Unified Method for Assessment of Clinical Feasibility
EHRA: European Heart Rhythm Association
ESS: Epworth Sleepiness Scale
ODI: Oxygen Desaturation Index
OSA: Obstructive sleep apnea
PAT: Peripheral Artery Tone
PPG: Photoplethysmography
PSG: Polysomnography
ROC: Receiver Operator Characteristics
SpO2: Saturation of Peripheral Oxygen
